# Dynamic change of neutrophil to lymphocyte ratio and hemorrhagic transformation after thrombolysis in stroke

**DOI:** 10.1186/s12974-016-0680-x

**Published:** 2016-08-26

**Authors:** Zhiliang Guo, Shuhong Yu, Lulu Xiao, Xin Chen, Ruidong Ye, Ping Zheng, Qiliang Dai, Wen Sun, Changsheng Zhou, Shuiping Wang, Wusheng Zhu, Xinfeng Liu

**Affiliations:** 1 Department of Neurology, Jinling Hospital, Medical School of Nanjing University, 305 E Zhongshan Rd, Nanjing, 210002 Jiangsu Province China; 2 Department of Neurology, Second Affiliated Hospital of Soochow University, Suzhou, 215004 China; 3 Department of Medicine, Royal Melbourne Hospital, University of Melbourne, Melbourne, Australia; 4 Department of Radiology, Jinling Hospital, Medical School of Nanjing University, Nanjing, 210002 China; 5 Department of Neurology, PLA 123 Hospital, 1052 Yanshan Road, Yuhui District, Bengbu, 233000 China

**Keywords:** Neutrophil to lymphocyte ratio (NLR), Ischemic stroke, Thrombolysis, Hemorrhagic transformation, Biomarker

## Abstract

**Background:**

The neutrophil to lymphocyte ratio (NLR) has been shown to predict short- and long-term outcomes in ischemic stroke patients. We sought to explore the temporal profile of the plasma NLR in stroke patients treated with intravenous thrombolysis (IVT) and its relationship with intracranial bleeding complications after thrombolysis.

**Methods:**

A total of 189 ischemic stroke patients were prospectively enrolled. Blood samples for leukocyte, neutrophil, and lymphocyte counts were obtained at admission and at 3–6, 12–18, and 36–48 h after IVT. Head CT was performed on admission and repeated after 36–48 h, and a CT scan was done immediately in case of clinical worsening. Hemorrhagic events were categorized as symptomatic intracranial hemorrhage (sICH) and parenchymal hematomas (PH) according to previously published criteria.

**Results:**

An increasing trend in the NLR was observed after stroke, and the NLR was higher in patients who developed PH or sICH at 3–6, 12–18, and 36–48 h after IVT (*P* < 0.01) than in those without PH or sICH. The optimal cutoff value for the serum NLR as an indicator for auxiliary diagnosis of PH and sICH was 10.59 at 12–18 h. Furthermore, the NLR obtained at 12–18-h post-treatment was independently associated with PH (adjusted odds ratio [OR] 1.14) and sICH (adjusted OR 1.14). In addition, patients with a NLR ≥10.59 had an 8.50-fold greater risk for PH (95 % confidence interval [CI] 2.69–26.89) and a 7.93-fold greater risk for sICH (95 % CI 2.25–27.99) than patients with a NLR <10.59.

**Conclusions:**

NLR is a dynamic variable, and its variation is associated with HT after thrombolysis in stroke patients.

**Electronic supplementary material:**

The online version of this article (doi:10.1186/s12974-016-0680-x) contains supplementary material, which is available to authorized users.

## Background

Multiple randomized controlled trials have demonstrated the efficacy of intravenous recombinant tissue plasminogen activator (IV rtPA) administered up to 4.5 h after the onset of symptoms of ischemic stroke [1]. However, the risk of hemorrhagic transformation (HT) is increased by as much as tenfold after IV rtPA, largely due to reperfusion injury and the toxic effects of rtPA [2]. In addition to the already known indicators for HT [2], the detection of new paradigms is still worthwhile. In addition, an improved understanding of the prevention or early risk assessment of rtPA-related HT may also be applicable to other reperfusion strategies such as endovascular therapy.

In animal studies, neutrophils have been shown to contribute to intracerebral hemorrhaging after treatment with rtPA following cerebral ischemia, while depletion of neutrophils reduces blood–brain barrier (BBB) disruption and the rate of HT [3, 4]. In humans, infiltration of matrix metalloproteinase-9 (MMP-9)-positive neutrophils is associated with BBB breakdown, basal lamina type IV collagen degradation, and HT [5]. Recent studies suggested that the initial neutrophil to lymphocyte ratio (NLR) is associated with mortality and infarct size in ischemic stroke [6, 7] and can predict the 90-day outcome after endovascular therapy [8]. However, all of these studies mainly focused on static NLR values at baseline, which may not reflect the comprehensive dynamic changes of patients’ conditions. Furthermore, there is also a lack of information on the clinical value of the NLR in acute ischemic stroke patients treated with IV rtPA, especially its relationship with the most serious and common complication of IV rtPA treatment, HT.

Thus, we aimed to explore the temporal variation of the NLR in patients and its relationship with the most serious subtypes of HT, namely symptomatic intracranial hemorrhage (sICH) and parenchymal hematoma (PH), in patients with ischemic stroke treated with IV rtPA [9].

## Methods

### Study population

Consecutive ischemic stroke patients admitted to the Departments of Neurology at two hospitals (Jinling Hospital and PLA 123 Hospital, both large comprehensive hospitals) from March 2012 to August 2015 were prospectively recruited. The inclusion criteria for enrollment were (1) age ≥18 years and (2) diagnosis of acute ischemic stroke and treatment with IV rtPA within 4.5-h post-onset. The study exclusion criteria were (1) evidence of active infection before admission or any systemic infection that occurred during the first 48 h after treatment with IV rtPA (41 patients); (2) cancer, chronic inflammation, autoimmune disease, or steroid therapy (6 patients); and (3) unavailability to complete blood cell count or medical records (8 patients discharged on the same day of admission). At last, 189 consecutive ischemic stroke patients were included in the current study. The study protocol was approved by the Institutional Human Research Ethics Committees of Jinling Hospital and PLA 123 Hospital, and all patients or their relatives gave informed consent.

### Treatment administration

IV rtPA (alteplase, 0.9 mg/kg up to a maximum of 90 mg/kg) was used with 10 % of the total dosage as a bolus, followed by a 60-min infusion of the remaining dose. Patients who were receiving a bridging therapy consisting of IV rtPA followed by endovascular therapy were also enrolled. The method of endovascular therapy, such as local intra-arterial thrombolysis using rtPA, mechanical thrombectomy, angioplasty, stent placement, or multimodal endovascular therapy, was left to the discretion of the neurointerventionists.

### Clinical protocol and laboratory tests

Patient’s medical history, including potential stroke risk factors, clinical examination findings, blood and coagulation test results, 12-lead electrocardiographs, and chest radiographs were obtained at admission. Stroke severity was assessed by a certified neurologist using the National Institutes of Health Stroke Scale (NIHSS) at admission and at 3–6, 12–18, and 36–48 h after treatment with IV rtPA. Neurological deterioration was defined as death or an increase of ≥4 points in the NIHSS score between the two examinations [10].

Venous blood samples were obtained from all patients at admission and at 3–6, 12–18, and 36–48 h after treatment with IV rtPA. Total leukocyte, neutrophil, and lymphocyte counts were determined using a COULTER LH780 Hematology Analyzer (Beckman Coulter, Inc, Orange County, CA). The NLR was calculated as the ratio of the percentage of neutrophils over the percentage of lymphocytes, both obtained from the same blood sample.

### CT and intracranial hemorrhage

On admission, all patients underwent a CT scan within the first 4.5 h of stroke onset. CT was repeated at 36–48 h, and another CT scan was done immediately in case of rapid neurological deterioration to evaluate the presence of HT. CT images were reviewed by a neuroradiologist with extensive experience in acute stroke who was blinded to patients’ medical records. PH was defined as hemorrhage with a mass effect according to previously published criteria [11]. sICH was defined as any hemorrhage in the brain on the CT scan accompanied by the presence of neurological deterioration [10].

### Statistical analysis

To compare baseline characteristics between groups according to the presence of PH or sICH, parametric and non-parametric comparisons were performed with the *t* test, *χ*^2^ test, and Mann-Whitney *U* test as appropriate. The relation of the NLR with two endpoints was investigated using logistic regression models. For multivariate analysis, we first included age and sex (model 1) and then additionally included variables that significantly correlated with PH or sICH in the univariate analysis (*P* < 0.10; models 2 or 3). Receiver operating characteristic (ROC) curves were used to test the overall discriminative ability of the NLR for PH or sICH and to establish optimal cutoff points at which the sum of the specificity and sensitivity was the highest. The differences in discriminative ability were tested using the DeLong method [12]. Finally, logistic regression analysis was performed again with the same independent variables as in the previous model, except for values of the NLR that were included as a binary variable according to the cutoff point. Statistical analysis was performed using SPSS for Windows, version 17.0 (SPSS Inc., Chicago, IL, USA) and SAS version 9.1 (SAS Institute Inc., Cary, NC). Two-tailed significance values were applied, and statistical significance was defined as *P* < 0.05.

## Results

### Baseline characteristics of patients

One hundred eighty-nine patients with ischemic stroke met the study criteria. The demographic and clinical characteristics between the included and excluded patients are detailed in Additional file 1: Table S1. These included cohorts from different hospitals are described in Additional file 1: Table S2. Among the 189 patients, 28 (14.8 %) presented with PH, and 17 (9.0 %) developed sICH. The mean time of sICH (determined by head CT) was 12.23 ± 7.74 h after thrombolysis. The main baseline characteristics of patients according to the presence or absence of PH or to the presence or absence of sICH are presented in Table 1.

**Table 1:** Baseline characteristics of patients according to the presence/absence of PH or sICH

	No PH (*n* = 161)	PH (*n* = 28)	*P*	No sICH (*n* = 172)	sICH (*n* = 17)	*P*
Age, years, mean (SD)	64.1 ± 10.3	70.1 ± 10.5	0.005	64.6 ± 10.4	68.7 ± 11.7	0.133
Females, %	59 (36.6)	7 (25.0)	0.233	60 (34.9)	6 (35.3)	0.973
Body mass index, kg/m^2^, mean (SD)	24.4 ± 3.1	23.9 ± 3.0	0.401	24.4 ± 3.0	24.0 ± 3.5	0.601
Hypertension, %	101 (62.7)	21 (75.0)	0.210	111 (64.5)	11 (64.7)	0.989
Diabetes, %	50 (31.1)	7 (25.0)	0.519	51 (29.7)	6 (35.3)	0.629
Hyperlipidemia, %	73 (45.3)	12 (42.9)	0.807	79 (45.9)	6 (35.3)	0.400
Previous stroke, %	14 (8.7)	5 (17.9)	0.251	17 (9.9)	2 (11.8)	1.000
Coronary artery disease, %	18 (11.2)	5 (17.9)	0.494	19 (11.0)	4 (23.5)	0.266
Atrial fibrillation, %	48 (29.8)	12 (42.9)	0.171	50 (29.1)	10 (58.8)	0.012
Current smokers, %	53 (32.9)	8 (28.6)	0.650	56 (32.6)	5 (29.4)	0.791
Ongoing antiplatelet therapy, %	9 (5.6)	7 (25.0)	0.002	12 (7.0)	4 (23.5)	0.060
SBP, mm Hg, mean (SD)	148.4 ± 18.4	154.5 ± 17.1	0.104	148.9 ± 18.2	153.2 ± 18.7	0.302
DBP, mm Hg, mean (SD)	81.4 ± 9.8	83.2 ± 10.4	0.368	81.3 ± 9.7	84.8 ± 11.2	0.163
Blood glucose, mmol/L, median (IQR)	7.2 (5.3–9.0)	7.0 (5.8–9.5)	0.133	7.0 (5.3–9.0)	8.0 (5.5–9.5)	0.645
Platelets, 10^9^/L, mean (SD)	187.9 ± 50.8	178.9 ± 40.0	0.371	187.3 ± 49.9	179.7 ± 44.5	0.545
INR, mean (SD)	1.01 ± 0.08	1.01 ± 0.09	0.468	1.01 ± 0.08	1.03 ± 0.08	0.302
Baseline NIHSS, median (IQR)	11 (6–15)	17 (10–22.5)	0.001	12 (6–15)	13 (10–20)	0.198
Onset to treatment, min, mean (SD)	170.6 ± 48.8	183.4 ± 51.9	0.207	171.1 ± 48.9	187.2 ± 52.1	0.198
IV rtPA + endovascular therapy, %	49 (30.4)	9 (32.1)	0.856	51 (29.7)	7 (41.2)	0.326

*DBP* diastolic blood pressure, *INR* international normalized ratio, *IQR* interquartile range, *IV rtPA* intravenous recombinant tissue plasminogen activator, *NIHSS* National Institutes of Health Stroke Scale, *PH* parenchymal hemorrhage, *SBP* systolic blood pressure, *SD* standard deviation, *sICH* symptomatic intracranial hemorrhage

### Temporal profile of NLR depending on the type of HT

**Fig. 1:**
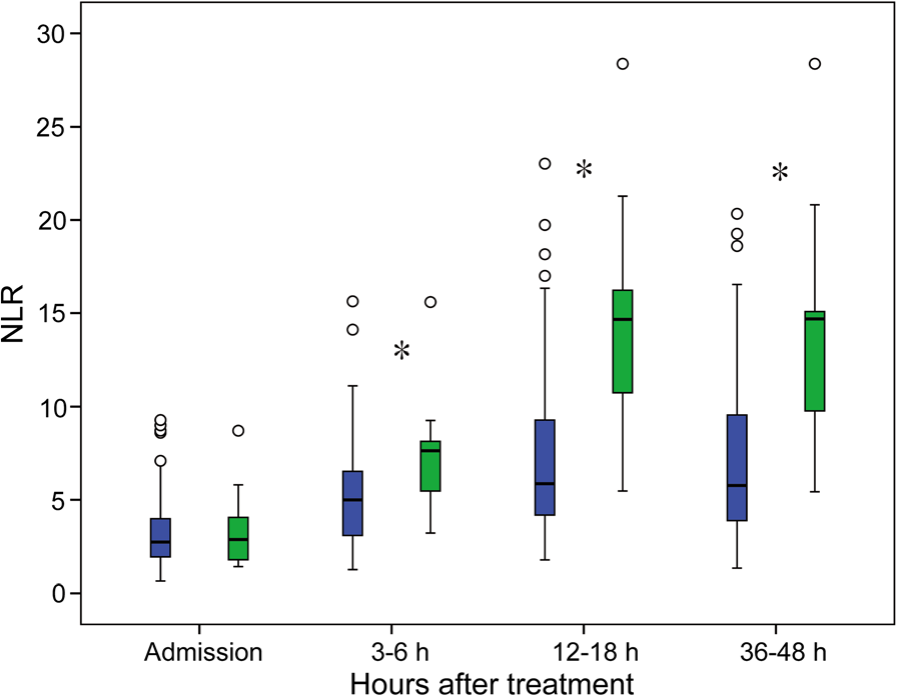
Temporal profile of plasma neutrophil to lymphocyte ratio (NLR) in stroke patients treated with recombinant tissue plasminogen activator (rtPA) according to the presence of parenchymal hemorrhage (PH). *Blue boxes* patients without PH, *green boxes* patients with PH. *P < 0.001 between patients with and without PH

**Fig. 2:**
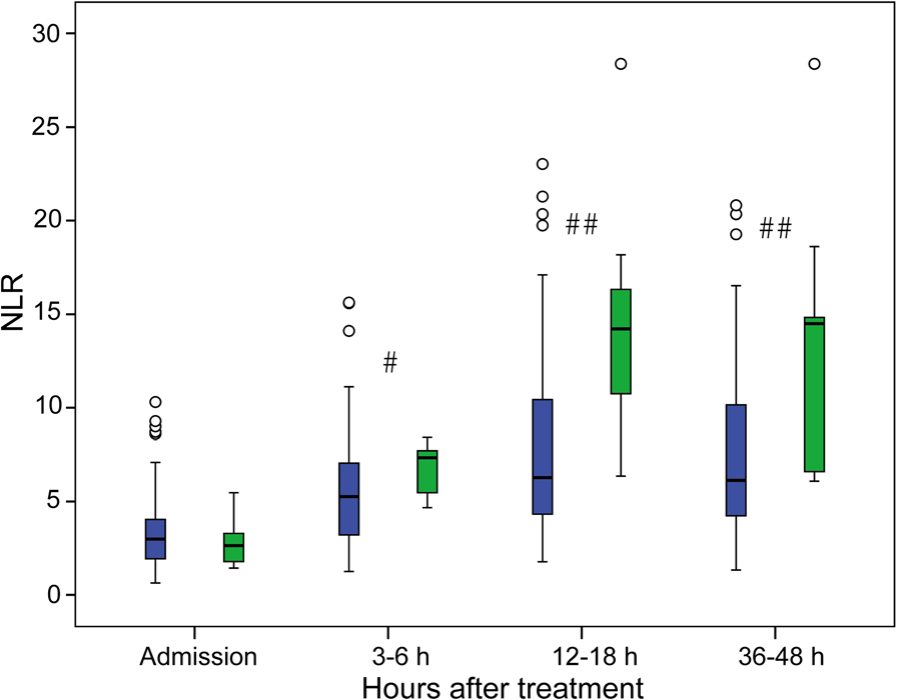
Temporal profile of plasma neutrophil to lymphocyte ratio (NLR) in stroke patients treated with recombinant tissue plasminogen activator (rtPA) according to the presence of symptomatic intracranial hemorrhage (sICH). *Blue boxes* patients without sICH, *green boxes* patients with sICH. ^#^ *P* = 0.009 between patients with and without sICH; ^##^ *P* < 0.001 between patients with and without sICH

The NLR was obtained at four different time points: at admission, 3–6 h after rtPA treatment, 12–18 h after rtPA treatment, and 36–48 h after rtPA treatment. The temporal profiles of the NLR according to the presence of PH or sICH are presented in Figs. 1 and 2 and Additional file 1: Table S3 and Figure S1. The NLR at admission did not differ between patients with and without PH (*P* = 0.819). Thereafter, an increasing trend in the NLR was observed in both groups (Additional file 1: Figure S1). However, the NLR values in the PH group were significantly higher than those in the No PH group at 3–6, 12–18, and 36–48 h after rtPA (*P* < 0.001). The temporal profile of the NLR according to the presence of sICH was similar to that according to the presence of PH.

### Association of plasma NLR with hemorrhagic transformation

**Fig. 3:**
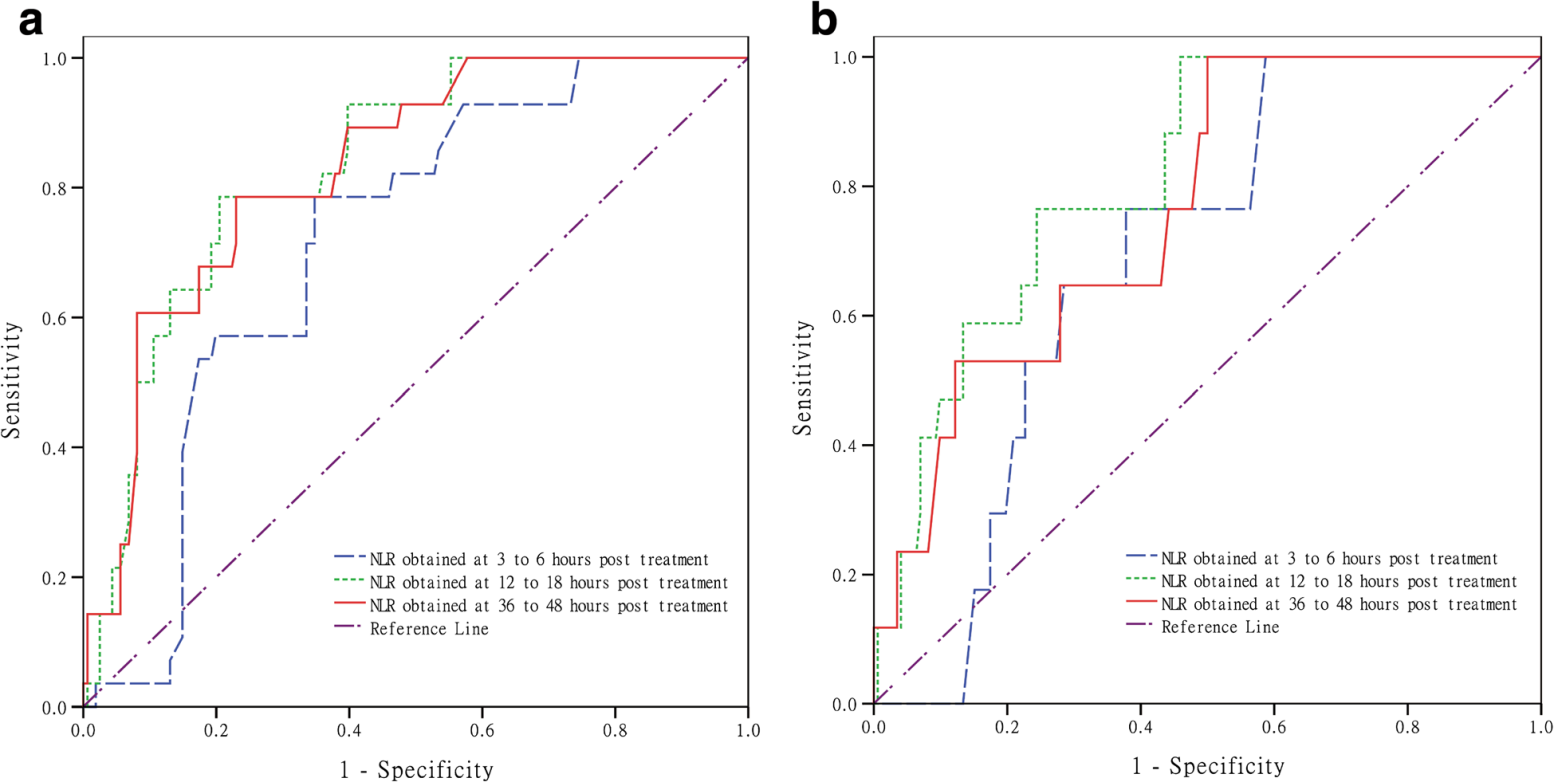
Discriminative ability of the neutrophil to lymphocyte ratio (NLR) for parenchymal hemorrhage (PH) and symptomatic intracranial hemorrhage (sICH). **a** Receiver operator characteristic (ROC) curve for NLR in auxiliary diagnosis of PH and **b** ROC curve for NLR in the auxiliary diagnosis of sICH

ROC curve analysis was performed to assess the best cutoff value of the NLR for discriminating PH and sICH (Fig. 3 and Table 2). The optimal cutoff value of the NLR that best distinguished the presence/absence of PH and sICH was 10.59 at 12–18 h after rtPA treatment, which can be obtained earlier than the NLR at 36–48 h after rtPA. The areas under the curve (AUCs) for the ability of the NLR to predict PH or sICH were 0.833 with 78.6 % sensitivity and 79.5 % specificity and 0.814 with 76.5 % sensitivity and 75.6 % specificity, respectively.

**Table 2:** Diagnostic values of the neutrophil to lymphocyte ratio (NLR) for PH and sICH

	AUC (95 % CI)	*P*	Cutoff value	Sensitivity (%)	Specificity (%)
For PH					
3–6 h NLR	0.717 (0.630–0.803)	<0.001	5.45	78.6	65.2
12–18 h NLR	0.833 (0.764–0.903)	Reference	10.59	78.6	79.5
36–48 h NLR	0.830 (0.758–0.902)	0.887	14.36	60.7	91.9
For sICH					
3–6 h NLR	0.691 (0.597–0.785)	<0.001	5.45	76.5	62.8
12–18 h NLR	0.814 (0.728–0.900)	Reference	10.59	76.5	75.6
36–48 h NLR	0.766 (0.666–0.866)	0.166	14.36	52.9	87.8

*AUC* area under the curve, *CI* confidence interval, *PH* parenchymal hematoma, *sICH* symptomatic intracranial hemorrhage

Table 3 summarizes the results of the binary logistic regression analysis of PH and sICH. The NLR as a continuous variable was independently associated with a greater risk of PH with an adjusted odds ratio (OR) of 1.17 (95 % CI, 1.10–1.26) with adjustment for age and sex (model 1) and 1.14 (1.05–1.23) with further adjustment for ongoing antiplatelet therapy and baseline NIHSS (model 2), respectively. In addition, age (adjusted OR 1.06, 95 % CI: 1.01–1.11; *P* = 0.014) and ongoing antiplatelet therapy (adjusted OR 4.00, 95 % CI: 1.13–14.14; *P* = 0.031) remained significant outcome predictors for PH. Furthermore, in our study, the risk of PH was also associated with NLR levels as a dichotomous variable (OR 8.50, 95 % CI: 2.69–26.89; *P* < 0.001) after adjustment for age, sex, ongoing antiplatelet therapy, and baseline NIHSS (model 2). This relationship was further confirmed in the dose-response model (Additional file 1: Figure S2). For the binary logistic regression analysis for sICH, similar associations were found between the NLR and sICH. A NLR level ≥10.59 (OR 7.93, 95 % CI 2.25–27.99; *P* = 0.001) remained significantly associated with sICH after adjusting for age, sex, atrial fibrillation, and ongoing antiplatelet therapy (model 3).

**Table 3:** Associations of the neutrophil to lymphocyte ratio (NLR) with PH and sICH

	PH OR (95 % CI)			sICH OR (95 % CI)		
	Univariate analysis	Model 1	Model 2	Univariate analysis	Model 1	Model 3
NLR^a^	1.19 (1.10–1.28)*	1.17 (1.10–1.26)*	1.14 (1.05–1.23)**	1.16 (1.08–1.26)*	1.16 (1.07–1.25)*	1.14 (1.06–1.23)**
NLR^b^ (≥10.59 vs <10.59)	14.22 (5.34–37.91)*	11.86 (4.35–32.36)*	8.50 (2.69–26.89)*	10.06 (3.11–32.52)*	9.55 (2.84–32.04)*	7.93 (2.25–27.99)**

*Model 1* bivariate logistic regression analyses with adjustment for age and sex. *Model 2* bivariate logistic regression analyses with adjustment for age, sex, ongoing antiplatelet therapy, and baseline NIHSS. *Model 3* bivariate logistic regression analyses with adjustment for age, sex, atrial fibrillation, and ongoing antiplatelet therapy. *CI* confidence interval, *OR* odds ratio, *PH* parenchymal hemorrhage, *sICH* symptomatic intracranial hemorrhage

*P < 0.001; **P < 0.05

^a^NLR as a continuous variable

^b^NLR as a dichotomous variable

## Discussion

This study first shows that the NLR is a dynamic variable, and its variation is associated with HT after treatment with IV rtPA in patients with acute stroke. In addition, the best discriminating value of the NLR for PH and sICH was 10.59 or more at 12–18-h post-treatment, which was associated with an 8.50-fold increased risk for PH and a 7.93-fold increased risk for sICH.

In previous studies, a high NLR was found to be independently associated with an increased risk of stroke in atrial fibrillation [13]. Moreover, the initial NLR is associated with infarct size and mortality rate in ischemic stroke [6, 7], and it also has predictive value for 90-day outcome after endovascular therapy [8]. However, the previous studies did not explore the clinical value of the NLR in acute ischemic stroke patients treated with IV rtPA.

Recently, Maestrini et al. reported that higher neutrophil counts and NLR before thrombolysis for cerebral ischemia are independently associated with sICH and worse outcome at 3 months [14]. However, they did not exclude patients with previous infections, which might contribute to the difference in the predictive values of the baseline NLR for sICH between their study and ours. That is because infections can lead to poor outcome after stroke via many different mechanisms, which may include (1) increased BBB disruption and tissue damage by neutrophil-derived various proteases, reactive oxygen species (ROS), as well as numerous inflammatory mediators; (2) impaired tissue reperfusion through endothelia-dependent mechanisms; (3) increased platelet activation and microvascular coagulation; and (4) CRP-induced ischemic tissue injury via a complement-dependent mechanism [15, 16]. Therefore, this shows that neutrophils are just one of many different mechanisms, and the association between the baseline NLR and endpoints may disappear after adjustment for infections. The reasons for such a difference may also relate to what the NLR represents. Changes observed in the baseline NLR could reflect the disease itself or external environment factors such as infection or cancer. Our results and their conclusions do not contradict per se because their study population is different to ours, with the NLR at baseline reflecting both the disease itself and the external environment factors in their study, but only the disease itself in our study.

The study by Maestrini et al. has a higher statistical power than our study based on the larger sample size [14], and we also believe that their results are fully credible and reliable. Higher numbers of baseline neutrophils at baseline have greater potential to induce tissue damage via the release of various proteolytic enzymes, ROS, and numerous inflammatory mediators [2]. Thus, in theory, patients with infection or other conditions that can potentially change baseline the NLR have a higher risk for the occurrence of HT after treatment with IV rtPA. Similarly, when we reintegrate the patients with infection or other conditions with potential to change the NLR into our analysis, the baseline NLR is higher in patients with HT (Additional file 1: Figure S3).

Moreover, the neutrophil and lymphocyte counts after ischemic stroke exhibit significant temporal variation [17], which is also indirectly confirmed by Maestrini et al. in their evaluation of the influence of the onset-to-sample time on the neutrophil count, leukocyte count, and NLR [14]. This suggests that the neutrophil count, lymphocyte count, and NLR are “dynamic” variables. However, Maestrini et al. mainly focused on static neutrophil count and NLR values at baseline [14], which may not have dynamically and comprehensively reflected the patients’ conditions. The clinical application value of dynamically testing the neutrophil count, lymphocyte count, and NLR in sICH and worse outcomes may be meaningful. Holding strict exclusion criteria in our study, we found that the NLR changed dynamically and a high NLR at 12–18 h after treatment with IV rtPA was independently associated with HT after IV rtPA. In addition, although there were no differences between the NLR at 12–18 h and the NLR at 36–48 h for auxiliary diagnosis of PH and sICH, we hold that the NLR at 12–18 h may be more valuable than that at 36–48 h. First, the NLR at 12–18 h could be obtained earlier than the NLR at 36–48 h, which could allow for better monitoring and could better reflect the severity and progression of disease, helping clinicians to adjust medication regimens and apply related auxiliary examination in time. Moreover, the NLR at 12–18 h was not inferior to the NLR at 36–48 h, and it exhibited a tendency to rise superior to the NLR at 36–48 h for diagnosing sICH. Therefore, we believe that the NLR at 12–18 h is the appropriate selection based on the main concerns in the present study.

The mechanisms underlying these observations are not well established, but they seem to be related to the roles of neutrophils and lymphocytes in ischemic insult and the disruption of the BBB. Circulating neutrophils are recruited to the site of cerebral injury shortly after ischemia occurs and then further contribute to BBB disruption and tissue damage via a variety of mechanisms [18–20]. Neutrophils have been shown to be an important source of MMP-9, which may open the BBB inside the lumen of the blood vessel by acting directly on tight junction proteins or may be absorbed into endothelial cells and act on the basement membrane [2]. In a rat cerebral ischemia model, treatments preventing neutrophil infiltration reduced MMP-9 released in the brain [21]. Moreover, inhibition or depletion of neutrophils can reduce the BBB breakdown and the rate of HT in ischemic stroke [3, 22]. In contrast, when neutrophils are increased via lipopolysaccharide or granulocyte colony-stimulating factor administration, there is an increase in BBB disruption in a mice model [23] and an increase in MMP-9 and rtPA-related HT in a rat stroke model [4]. In humans with ischemic stroke, early neutrophilia is associated with larger infarct volumes [24], and MMP-9-positive neutrophil infiltration has also been associated with disruption of the BBB, basal lamina type IV collagen degradation, and HT [5]. Thus, neutrophils may mediate HT through neutrophil-derived MMPs in ischemic stroke. In addition to neutrophil-derived MMPs, other factors released from neutrophils after stroke [20], including ROS, myeloperoxidase, elastase, cathepsin G, proteinase 3, cytokines, and chemokines, can also disrupt the neurovascular unit and ultimately result in increased BBB permeability and HT [17].

In addition to the mechanisms mediated by factors released from neutrophils, novel aspects of neutrophil biology may also contribute to ischemic brain injury. Recently, activated neutrophils have been described to form neutrophil extracellular traps (NETs), a web-like structure composed of DNA, histones, and specific granule proteins, such as neutrophil elastase and myeloperoxidase, in response to various stimuli [25]. Recent evidence indicates that a lack of NETs during myocardial and liver ischemia/reperfusion (I/R) injury offers significant cardioprotective, hepatoprotective, and anti-inflammatory effects [26, 27]. Furthermore, extracellular chromatin and histones exacerbate cerebral I/R injury in mice [28]. These results suggest that NETs may play a role in BBB disruption and tissue damage. Further studies are needed to explore whether NETs have deleterious effects on HT.

The lymphocyte counts might serve as an index for general health, influenced by acute physiologic stress [29]. Relative lymphopenia on the other hand reflects the cortisol-induced stress response and sympathetic tone [30], which can increase the production of proinflammatory cytokines that aggravate ischemic injury [31]. This means that low lymphocyte counts in patients with HT are not merely an initial response to severe stroke, but that lymphocytes may be actively involved in a protective mechanism in the ischemic brain. Experimental evidence suggests that specific subtypes of lymphocytes (namely, regulatory T cells) play key roles in abrogating the inflammatory response and are major cerebroprotective immunomodulators in acute stroke [32]. Our findings suggest that lower lymphocyte counts in patients with HT (data not shown) might have been the result of fewer regulatory T cells being available to curtail the inflammatory response, thereby leading to greater tissue damage. However, other subtypes of lymphocytes (namely, proinflammatory lymphocytes) may have a deleterious effect on I/R injury [33]. It is uncertain which subtype of lymphocytes has a dominant role in the pathophysiology of cerebral ischemia, and we demonstrated that a decrease in lymphocytes as a whole has a negative effect on HT. Further studies are also needed to elucidate the complex immunomodulatory interactions that occur after stroke.

The NLR reflects the balance between neutrophil and lymphocyte levels, which may be comprehensively represent the immunological conditions. In this sense, the NLR is superior to only the neutrophil count or lymphocyte count for distinguishing the occurrence of HT, and this may also explain why the AUC for the NLR appeared to be greater than those for neutrophil and lymphocyte counts at each corresponding time point (Additional file 1: Table S4). On the other hand, inflammatory cytokines released by neutrophils may trigger lymphocyte apoptosis [34]. This suggests that the NLR may not simply reflect the neutrophil and lymphocyte counts but also overactivation of neutrophils, thus leading to a wider gap between the two leukocyte types, and this also supports the superiority of the NLR.

The main strength of our study is that the clinical information and blood samples taken at different time points from all patients were collected in a prospective fashion with strict exclusion criteria. Previous infections and early hospital infections in stroke are associated with an increase in leukocytes and poor outcomes [14, 35]. We limited these potential confounders by ruling out patients with infection. In addition, our observations of elevated neutrophil counts in patients with HT (data not shown) may offer a partial explanation as to why stroke patients with infection may have poor outcomes. Nonetheless, our current findings also have some limitations. First, the small sample size weakens the statistical strength of our conclusions. Therefore, further studies with larger samples are needed. Second, the study population included patients receiving a bridging strategy of the use of IV rtPA followed by endovascular therapy, which may interfere with our results. Nevertheless, similar results were found for patients experiencing a bridging strategy even if the sample was small (data not shown). On the other hand, this shows that the NLR may also be applicable to distinguish the presence and absence of HT in other reperfusion strategies such as endovascular therapy. Regrettably, a control group that did not experience reperfusion therapy was not included in our study. Third, we neither explored the mechanisms by which neutrophils and lymphocytes affect the BBB breakdown and HT nor investigated what factors regulate the dynamic changes in neutrophil and lymphocyte counts after ischemic stroke in animal studies. These will be the focus of our next work.

## Conclusions

In conclusion, our results suggest that the NLR is a dynamic variable and its variation is associated with the occurrence of HT after thrombolysis in patients with stroke.

## Additional file

Additional file 1: Tables S1 to S3 and Figures S1 to S4. (PDF 461 kb)

## Abbreviations

AUC: Area under the curve
BBB: Blood–brain barrier
CI: Confidence interval
DBP: Diastolic blood pressure
HT: Hemorrhagic transformation
I/R: Ischemia/reperfusion
INR: International normalized ratio
IQR: Interquartile range
IV rtPA: Intravenous recombinant tissue plasminogen activator
IVT: Intravenous thrombolysis
MMP-9: Matrix metalloproteinase-9
NETs: Neutrophil extracellular traps
NIHSS: National Institutes of Health Stroke Scale
NLR: Neutrophil to lymphocyte ratio
OR: Odds ratio
PH: Parenchymal hematoma
ROC: Receiver operating characteristic
ROS: Reactive oxygen species
SBP: Systolic blood pressure
SD: Standard deviation
sICH: Symptomatic intracerebral hemorrhage
