# Supplementary material for: Dynamic change of neutrophil to lymphocyte ratio and hemorrhagic transformation after thrombolysis in stroke
Source: J Neuroinflammation. 2016 Aug 26;13(1):199. doi: 10.1186/s12974-016-0680-x (PMC5000487; doi:10.1186/s12974-016-0680-x)
Supplement: Additional file 1: — Tables S1 to S3 and Figures S1 to S4. (PDF 461 kb) [file 12974_2016_680_MOESM1_ESM.pdf]

ONLINE SUPPLEMENT

Dynamic Change of Neutrophil to Lymphocyte Ratio and Hemorrhagic Transformation after Thrombolysis in Stroke

Supplemental Table I. Demographic and Clinical Characteristics of Included and Excluded Patients

Characteristics	Patients included	Patients excluded	*P*
No. of patients	189	55	
Age,years, mean (SD)	65.0±10.6	66.2±12.5	0.467
Female, (%)	66 (34.9)	20 (36.4)	0.844
Body mass index, kg/m^2, mean (SD)	24.3±3.1	24.4±3.3	0.926
Hypertension, (%)	122 (64.6)	40 (72.7)	0.259
Diabetes, %	57 (30.2)	18 (32.7)	0.716
Hyperlipidemia, %	85 (45.0)	22 (40.0)	0.513
Previous stroke, %	19 (10.1)	8 (14.5)	0.350
Coronary artery disease, %	23 (12.2)	9 (16.4)	0.417
Atrial fibrillation, %	60 (31.7)	21 (38.2)	0.372
Current smoker, %	61 (32.3)	14 (25.5)	0.335
Ongoing antiplatelet therapy, %	16 (8.5)	7 (12.7)	0.341
Baseline NIHSS, median (IQR)	12 (6-16)	14 (11-19.5)	0.004
Onset to treatment, min, mean (SD)	173.0±49.6	178.9±44.6	0.423
IV rtPA + endovascular therapy, %	58 (30.7)	20 (36.4)	0.427

IV rtPA indicates intravenous recombinant tissue plasminogen activator; NIHSS, National Institutes of Health Stroke Scale; SD, standard deviation.

Supplemental Table II. Demographic and Clinical Characteristics of Included Patients from Different Hospitals

Characteristics	Jinling Hospital	PLA 123 Hospital	*P*
No. of patients	105	84	
Age,years, mean (SD)	65.4±9.8	64.5±11.5	0.467
Female, (%)	37 (35.2)	29 (34.5)	0.918
Body mass index, kg/m^2, mean (SD)	24.6±3.1	24.1±3.0	0.926
Hypertension, (%)	72 (68.6)	50 (59.5)	0.196
Diabetes, %	28 (26.7)	29 (34.5)	0.242
Hyperlipidemia, %	56 (43.8)	22 (46.4)	0.719
Previous stroke, %	12 (11.4)	7 (8.3)	0.482
Coronary artery disease, %	12 (11.4)	11 (13.1)	0.728
Atrial fibrillation, %	34 (32.4)	26 (42.9)	0.834
Current smoker, %	37 (35.2)	24 (28.6)	0.330
Ongoing antiplatelet therapy, %	9 (8.6)	7 (8.3)	0.953
Baseline NIHSS, median (IQR)	12 (7-17)	11 (6-15)	0.306
Onset to treatment, min, mean (SD)	173.6±51.4	171.2±46.8	0.423
IV rtPA + endovascular therapy, %	30 (28.6)	28 (33.3)	0.481
PH, %	15 (14.3)	13 (15.5)	0.819
sICH, %	9 (8.6)	8 (9.5)	0.820

IV rtPA indicates intravenous recombinant tissue plasminogen activator; NIHSS, National Institutes of Health Stroke Scale; PH, parenchymal hemorrhage; SD, standard deviation; sICH, symptomatic intracranial hemorrhage.

Supplemental Table III. Temporal Profile of Plasma Neutrophil to Lymphocyte Ratio (NLR) in Stroke Patients Treated with rtPA Regarding the Presence of PH or sICH

	No PH	PH	No sICH	sICH
Admission NLR	2.73 (1.91-4.00)	2.86 (1.78-4.13)	2.99 (1.94-4.05)	2.64 (1.67-3.34)
3-6 h NLR	4.99 (3.09-6.62)	7.64 (5.47-8.17)*	5.26 (3.21-7.05)	7.35 (5.07-7.72)[#]
12-18 h NLR	5.86 (4.18-9.28)	14.66 (10.75-16.28)*	6.28 (4.27-10.43)	14.22 (8.68-17.25)[##]
36-48 h NLR	5.77 (3.74-9.57)	14.69 (9.77-15.21)*	6.13 (4.23-10.15)	14.50 (6.48-16.72)[##]

PH indicates parenchymal hemorrhage; sICH, symptomatic intracranial hemorrhage. * indicates $P < 0.001$ compared with the No PH group. [#] indicates $P=0.009$ compared with the No sICH group, [##] indicates $P < 0.001$ compared with the No sICH group.

Supplemental Table IV. Values of the Plasma Neutrophil Level, Lymphocyte Level and Neutrophil to Lymphocyte Ratio (NLR) Level in auxiliary diagnosis of PH and sICH

	PH			sICH		
	AUC	95% CI	*P*	AUC	95% CI	*P*
Time I						
Neutrophils	0.569	0.449-0.689	0.243	0.524	0.395-0.654	0.740
Lymphocytes	0.441	0.327-0.554	0.318	0.520	0.402-0.637	0.789
NLR	0.514	0.395-0.632	0.819	0.419	0.299-0.540	0.274
Time II						
Neutrophils	0.682	0.596-0.769	0.002	0.726	0.632-0.821	0.002
Lymphocytes	0.285	0.199-0.371	< 0.001	0.323	0.230-0.415	0.016
NLR	0.717	0.630-0.803	< 0.001	0.691	0.597-0.785	0.009
Time III						
Neutrophils	0.797	0.711-0.884	< 0.001	0.805	0.705-0.906	< 0.001
Lymphocytes	0.169	0.102-0.237	< 0.001	0.176	0.089-0.264	< 0.001
NLR	0.833	0.764-0.903	< 0.001	0.814	0.728-0.900	< 0.001
Time IV						

Neutrophils	0.763	0.677-0.849	< 0.001	0.729	0.629-0.829	< 0.001
Lymphocytes	0.167	0.096-0.237	< 0.001	0.229	0.127-0.331	< 0.001
NLR	0.830	0.758-0.902	< 0.001	0.766	0.666-0.866	< 0.001

AUC indicates area under curve; PH, parenchymal hematomas; sICH, symptomatic intracranial hemorrhage; I, II, III and IV, on behalf of their respective admission and 3-6, 12-18, 36-48 hours after treatment with intravenous rtPA.

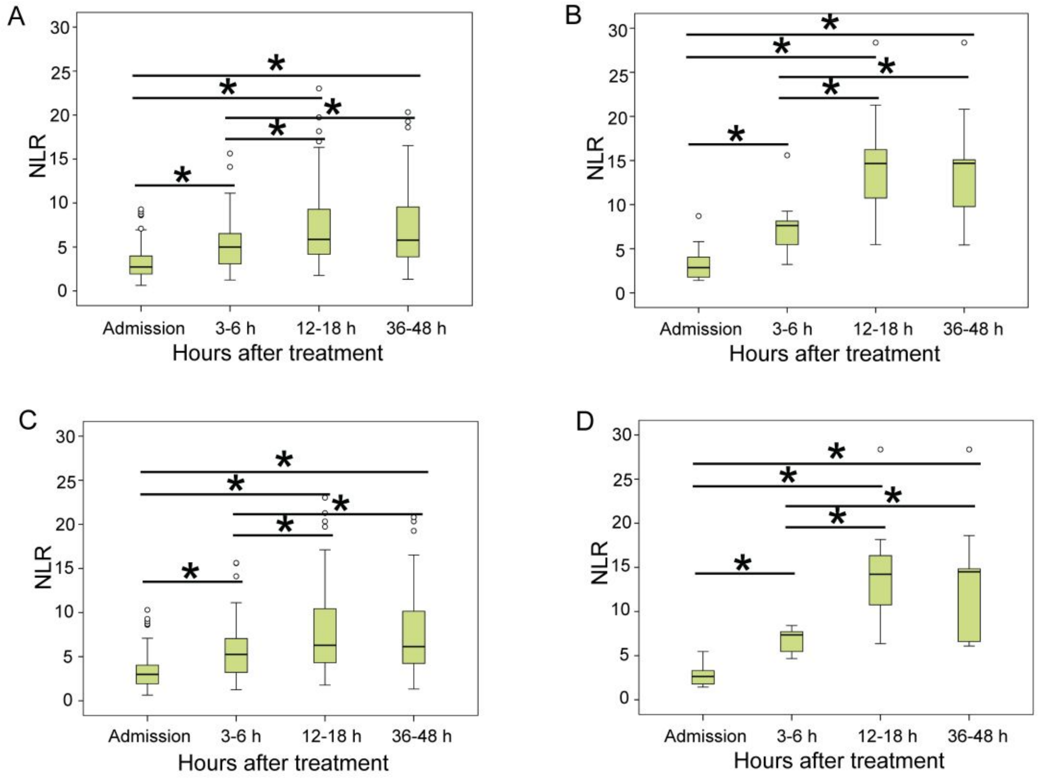

Supplemental Figure I. Temporal profile of plasma Neutrophil to Lymphocyte Ratio (NLR) in stroke patients treated with rtPA regarding the presence of parenchymal hemorrhage (PH) or the presence of symptomatic intracranial hemorrhage (sICH)

(A) temporal profile of plasma NLR in No PH; (B) temporal profile of plasma NLR in PH; (C) temporal profile of plasma NLR in No sICH; (D) temporal profile of plasma NLR in sICH. * $P < 0.001$.

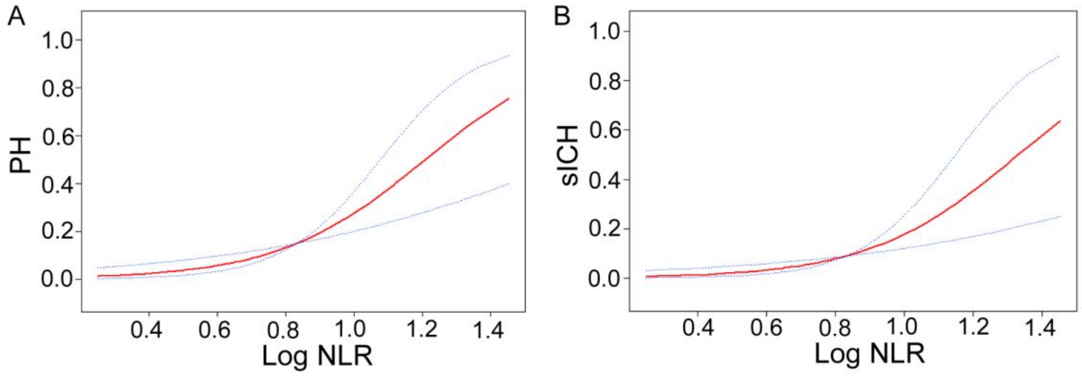

Supplemental Figure II. Plasma Neutrophil to Lymphocyte Ratio (NLR) level and hemorrhagic transformation dose-response relationship. (A) NLR and parenchymal hemorrhage (PH); adjusted for age, sex, ongoing antiplatelet therapy and baseline NIHSS. (B) NLR and symptomatic intracranial hemorrhage (sICH); adjusted for age, sex, atrial fibrillation and ongoing antiplatelet therapy. The analyses were performed using Empower(R) (www.empowerstats.com, X&Y solutions, inc. Boston MA) and R (http://www.R-project.org) (Environmental research. 2013;121:79-83). The NLR was transformed to the Log10 scale (Log NLR) for analysis.

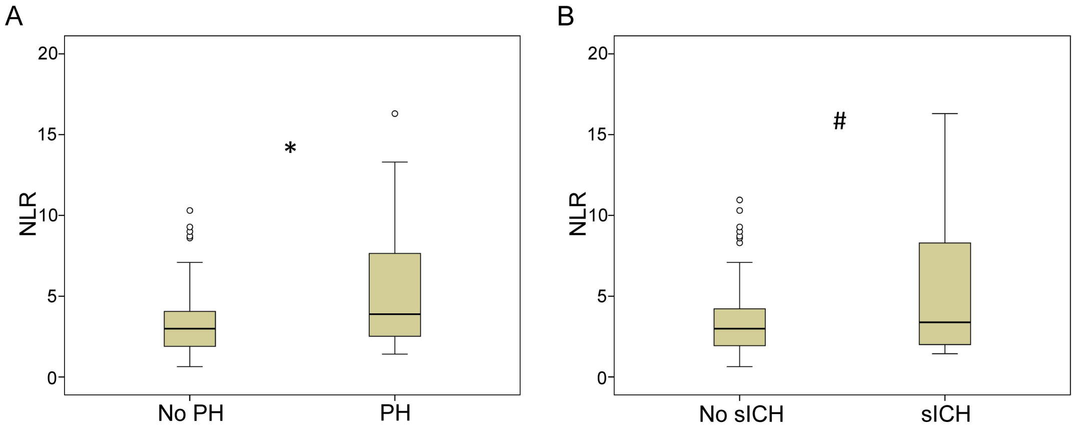

Supplemental Figure III. The levels of baseline Neutrophil to Lymphocyte Ratio (NLR) according to the presence of hemorrhagic transformation in all patients including those patients with infection or other conditions. (A) The levels of baseline NLR regarding the presence of parenchymal hemorrhage (PH); (B) The levels of baseline NLR regarding the presence of symptomatic intracranial hemorrhage (sICH). * P=0.002 between patients with and without PH; $^{\#}$ P=0.046 between patients with and without sICH.
